# The role of endosomal cholesterol trafficking protein, StAR-related lipid transfer domain 3 (StarD3/MLN64), in BRIN-BD11 insulinoma cells

**DOI:** 10.1007/s13238-016-0315-0

**Published:** 2016-09-27

**Authors:** Joana Borges Pinto, Annette Graham

**Affiliations:** 1 Department of Life Sciences, School of Health and Life Sciences, Glasgow Caledonian University, Glasgow, G4 0BA UK; 2 WolfsonWohl Cancer Research Centre, Institute of Cancer Sciences, University of Glasgow, Switchback Road, Bearsden, Glasgow, G61 1QH UK

**Dear Editor,**

Insulin secretion by pancreatic β-cells is modulated by altering the cellular content and distribution of cholesterol, which is tightly regulated by a network of transcription factors, enzymes, receptors, transporters and cholesterol trafficking proteins. Inhibition of cellular biosynthesis by ‘statin’ drugs, or depletion of plasma membrane cholesterol by methyl-β-cyclodextrin (MCD), reduce glucose-stimulated insulin secretion (GSIS) and content in β-cells and islets; statin treatment also causes delayed ATP production, inflammation and β-cell apoptosis (Tscuhiya et al., 2010; Zuniga-Hertz et al., 2015). Paradoxically, accumulation of β-cell/islet cholesterol caused by low density lipoprotein (LDL) receptor deficiency (Souza et al., 2013) or loss of ATP binding cassette transporters (ABCA1, ABCG1) which efflux cholesterol to apolipoprotein A-I (apo A-I) and high density lipoprotein (HDL) (Kruit et al., 2012), is also associated with loss of GSIS, impaired calcium handling, increased reactive oxygen species (ROS), inflammation and apoptosis.

Cholesterol levels within β-cells must therefore remain within defined limits to maintain insulin release. Sustaining or improving the efficiency of non-vesicular intracellular cholesterol transport, by targeting key members of the steroidogenic acute regulatory protein (StAR)-related lipid transfer (START) domain family, may help achieve this goal. The START domain of 54kDa endosomal StarD3 (MLN64) is an helix grip fold, providing a hydrophobic binding site for one molecule of cholesterol, facilitating cholesterol trafficking to the endoplasmic reticulum (ER), mitochondria and plasma membrane (Charman et al., 2010; Alpy et al., 2013; van der Kant et al., 2013). Our previous work demonstrated regulation of StarD3 expression by lipid-responsive transcription factors and macrophage sterol content (Borthwick et al. 2009) and repression by genetic obesity in hepatic tissues (Soffientini et al., 2014), while overexpression of STARD3 in macrophages enhanced expression of ABCA1 and cholesterol efflux to apoA-I (Borthwick et al., 2010).

Together, these data led to the hypothesis that StarD3 might be an important component of the cholesterol homeostasis mechanisms sustaining effective insulin release in β-cells. This study examines expression of StarD3 after cholesterol enrichment and depletion, and investigates the functional impact of StarD3 ligation and of genetically manipulating expression levels of this protein, on cholesterol metabolism and insulin release in rodent BRIN-BD11insulinoma cells.

A commercial Cholesterol Lipid Concentrate (CLC), containing cholesterol bound to cyclodextrin, was used to increase the cholesterol content of BRIN-BD11 insulinoma cells. The effects of treatment (1 h) with dilutions (1:250, 1:200 and 1:100) of CLC, followed by incubation for 24h in serum-free media, on cell viability, cholesterol biosynthesis and mass, insulin secretion and expression of StarD3 are shown in Table 1. Treatment with CLC did not reduce cellular viability; instead dilutions of 1:200 and 1:100 were associated with modest (14%; *P* < 0.01) increases in conversion of MTT to formazan. Cellular cholesterol mass increased by 50% (*P* < 0.001) at CLC (1:200), but no changes in cholesterol biosynthesis were noted at any dilution of CLC tested. Release of insulin into Krebs buffer containing 5.6 mmol/L glucose (20 min) increased (1.48-fold; *P* < 0.05) following treatment with CLC (1:100), but no significant changes in expression of StarD3 protein were noted at any dilution tested (Table 1).

**Table 1:** Effects of treatment with CLC, MCD and lutein on viability, cholesterol biosynthesis and/or efflux and mass, insulin release and expression of StarD3 protein in BRIN-BD11 cells

Treatment condition	Viability Formazan (μmol/L)	Cholesterol biosynthesis dpm/mg protein	Cholesterol efflux (%)	Cholesterol mass mg/mg protein	Insulin release (ng/mL)	Ratio of StarD3/Gapdh protein
Control	47.7 ± 8.08 (*n =* 4)	5542 ± 1171 (*n =* 4)	-	1.71 ± 0.40 (*n =* 6)	0.29 ± 0.049 (*n =* 4)	0.571 ± 0.298 (*n =* 3)
CLC 1:250	53.2 ± 14.7 (*n =* 4)	7478 ± 384 (*n =* 4)	-	1.90 ± 0.49 (*n =* 6)	0.31 ± 0.045 (*n =* 4)	0.524 ± 0.2376 (*n =* 3)
CLC 1:200	54.8 ± 6.02 (*n =* 4)*	5359 ± 1385 (*n =* 4)	-	2.56 ± 0.56 (*n =* 6)***	0.34 ± 0.043 (*n =* 4)	0.560 ± 0.229 (*n =* 3)
CLC 1:100	54.4 ± 7.74 (*n =* 4)*	7376 ± 2614 (*n =* 4)	-	1.42 ± 0.40 (*n =* 6)	0.43 ± 0.029 (*n =* 4)*	0.468 ± 0.25 (*n =* 3)
Control	47.6 ± 8.89 (*n =* 4)	8025 ± 2197 (*n =* 3)	0.67 ± 0.24 (*n =* 4)	1.62 ± 0.27 (*n =* 7)	0.25 ± 0.02 (*n =* 4)	0.32 ± 0.15 (*n =* 4)
MCD 0.1 mmol/L	47.6 ± 8.57 (*n =* 4)	7991 ± 2416 (*n =* 3)	0.53 ± 0.23 (*n =* 4)	1.33 ± 0.32 (*n =* 7)	0.17 ± 0.01 (*n =* 4)*	0.43 ± 0.18 (*n =* 4)
MCD 1 mmol/L	50.5 ± 8.09 (*n =* 4)	12101 ± 1621 (*n =* 3)	1.53 ± 0.98 (*n =* 4)	1.49 ± 0.38 (*n =* 7)	0.22 ± 0.02 (*n =* 4)	0.58 ± 0.30 (*n =* 4)
MCD 3 mmol/L	49.3 ± 7.44 (*n =* 4)	51294 ± 3517 (*n =* 3)	28.8 ± 3.30 (*n =* 4)***	0.51 ± 0.14 (*n =* 7)**	0.24 ± 0.04 (*n =* 4)	-
MCD 10 mmol/L	52.8 ± 5.51 (*n =* 4)	170890 ± 73394 (*n =* 3)**	89.7 ± 1.60 (*n =* 4)***	0.79 ± 0.17 (*n =* 7)**	0.14 ± 0.03 (*n =* 4)**	0.29 ± 0.12 (*n =* 4)
Control	242 ± 28.2 (*n =* 3)	6477 ± 708 (*n =* 3)	3.14 ± 0.75 (*n =* 3)	0.906 ± 0.157 (*n =* 3)	Fig. 1C	-
+ ApoAI (10 μg/mL)		-	4.71 ± 1.3 (*n =* 3)**			
Lutein 3 μg/mL + ApoA	246 ± 16.6 (*n =* 3)	- -	3.01 ± 0.68 (*n =* 3) 4.09 ± 1.1 (*n =* 3)*	0.856 ± 0.07 (*n =* 3)	-	-
Lutein 10 μg/mL + ApoAI (10 μg/mL)	236.9 ± 27.9 (*n =* 3)	8144 ± 541 (*n =* 3) -	3.20 ± 0.69 (*n =* 3) 3.93 ± 1.32 (*n =* 3)	0.862 ± 0.087 (*n =* 3)	Fig. 1C	-
Lutein 30 μg/mL + ApoAI (10 μg/mL)	230.3 ± 37.4 (*n =* 3)	- -	3.16 ± 0.56 (*n =* 3) 3.54 ± 1.2 (*n =* 3)	1.156 ± 0.098 (*n =* 3)	-	-

*P < 0.05; **P < 0.01; ***P < 0.001

Cholesterol depletion from BRIN-BD11 cells was achieved by treatment (1 h) with methyl-β-cyclodextrin (MCD; 0–10mmol/L), followed by a 24 h recovery period in serum-free media. No significant changes in cellular viability, as judged by production of formazan were observed (Table 1). Cholesterol mass decreased by 51% (*P* < 0.01) in cells treated with 10mmol/L MCD, reflected in the marked compensatory increase (20.8-fold; *P* < 0.01) in cholesterol (20.8-fold; *P <* 0.01) biosynthesis from [^14^C]acetate. In cells radiolabelled with [^3^H]cholesterol, 1mmol/L MCD and 10 mmol/L MCD significantly increased % cellular radiolabel extracted by 43.1-fold (*P* < 0.001) and by 134.7-fold (*P <* 0.001) respectively, compared with control. Insulin release (20min) into Krebs buffer (5.6 mmol/L glucose) declined by 33% (*P* < 0.05) and 45% (*P* < 0.01) after treatment with 0.1mmol/L and 10mmol/L MCD, respectively (Table 1). Levels of StarD3 protein fluctuated in cells treated with MCD, but the changes did not prove significant (Table 1), and linear regression analysis indicated no significant correlations with insulin release, efflux biosynthesis, or cholesterol mass. Together, these data-sets indicate that expression of StarD3 protein remain unaffected by profound changes in flux between cellular cholesterol pools induced by CLC and MCD in BRIN-BD11 insulinoma cells.

Treatment of the BRIN-BD11 cells with lutein (0–30 μmol/L), a carotenoid ligand of StarD3, did not significantly alter viability (Table 1) or induce any significant changes in StarD3 protein levels (Fig. 1A), compared with control, indicating ligation does not change StarD3 protein stability. However, levels of StarD3 protein were higher in cells incubated in serum-containing medium compared with those incubated under serum-free conditions, except in the presence of 30 μmol/L lutein. No significant changes in total cholesterol content or biosynthesis of [^14^C]cholesterol from [^14^C]acetate (Table 1) occurred following lutein treatment. Efflux of [^3^H]cholesterol to apoA-I (10 μg/mL), although modest, was significantly higher than that in the absence of any cholesterol acceptor; however, this significance was lost in cells exposed to 10–30 μmol/L lutein (Table 1). Efflux of [^3^H]cholesterol to HDL (50 μg/mL) was higher than to apoA-I: modest inhibitions of [^3^H]cholesterol efflux were noted at 3 μmol/L lutein (15%; *P <* 0.05) and 10 μmol/L lutein (16%; *P <* 0.01) (Fig. 1B). Release of insulin was not significantly increased by prior incubation with HDL (50 μg/mL; 24 h) under basal conditions, but in the presence of 10 μmol/L lutein, the stimulatory effect of HDL (50 μg/mL) proved significant (1.43-fold; *P <* 0.05) (Fig. 1C).

**Figure 1:**
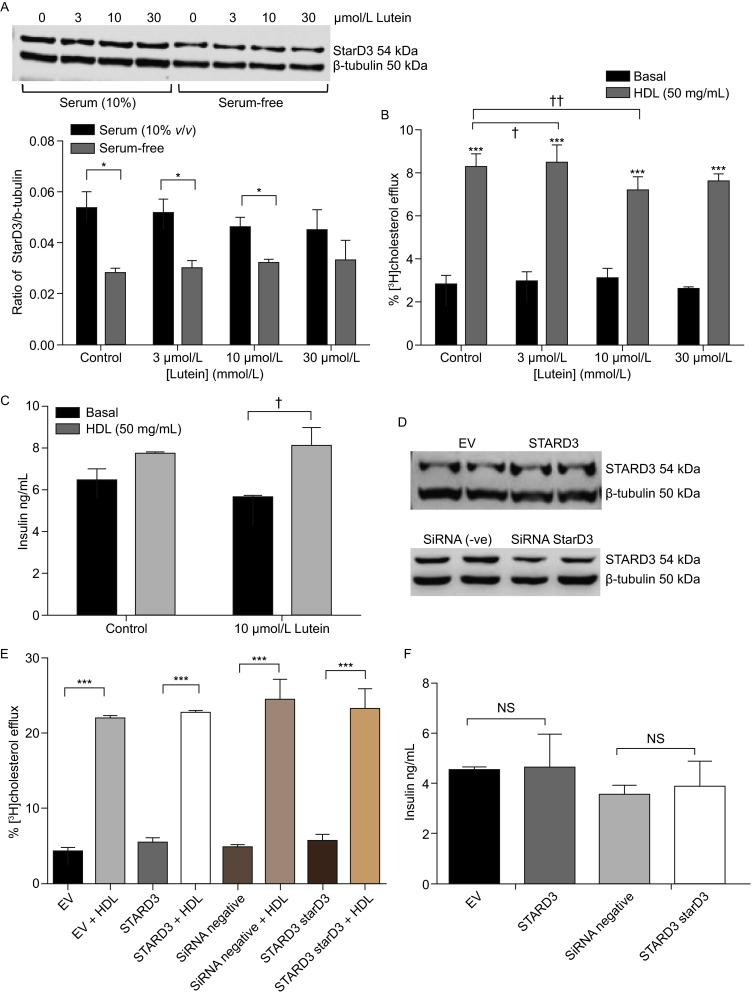
**The effect of ligation, and genetic manipulation, of StarD3 on levels of StarD3 protein, cholesterol efflux to HDL and insulin release by BRIN-BD11 cells**. (A) Levels of StarD3 protein, relative to the housekeeping protein β-tubulin, were measured in cells treated with lutein (0–30 μmol/L) in media containing 10% FBS (*v*/*v*) or under serum-free conditions. Repeated measures ANOVA (*P* = 0.0187) indicated differences between treatment conditions; significant differences were noted in Bonferroni post-tests between cells incubated in serum-free and serum-containing media, as indicated (*P < 0.05). Efflux of [^3^H]cholesterol in the presence of absence of HDL (50 μg/mL) (1B) and lutein (0–30 μmol/L) indicated differences between treatment conditions (repeated measures ANOVA *P <* 0.0001); Bonferroni post-tests indicated significant differences (***P < 0.001) compared with the basal condition, and with the vehicle control (^**†**^ *P <* 0.05;^**††**^ *P <* 0.01) in three separate experiments. Insulin release (1C) was measured in presence and absence of HDL (50 μg/mL) and lutein (10 μmol/L); repeated measures ANOVA (*P* = 0.0032) indicated differences between conditions; Bonferroni post-test indicated significance from the basal condition (*P < 0.01) and from lutein alone (^†^ *P <* 0.05) as shown. (D) Cells stably expressing the empty vector (EV) or STARD3, exhibited efflux of [^3^H]cholesterol to HDL (50 μg/mL) from cells stably expressing the empty vector (EV) or StarD3 (1E) that were significantly different in both cell lines in the presence of HDL (repeated measures ANOVA *P* = 0.0384; Bonferroni post-test *P < 0.05; *n =* 3) as indicated. Cholesterol efflux to HDL (50 μg/mL) from cells stably expressing the silencing plasmid control, or targeting StarD3 (1E) also showed differences between treatment conditions (repeated measures ANOVA *P <* 0.001); significance (*P < 0.001) in a Bonferroni post-test are indicated. Insulin release, in the presence of 50 μg/mL HDL, from cells overexpressing STARD3, or which have undergone StarD3 knockdown is shown in 1F: in a paired *t*-test (two-tailed), no significant differences were noted between EV and STARD3 (*P* = 0.8868) cells, or between the silencing plasmid targeting StarD3 and its control (*P* = 0.5387; *n =* 3)

Finally, BRIN-BD11 cells were genetically manipulated so that STARD3 was stably overexpressed or StarD3 stably repressed (Fig. 1D). Compared with empty vector (pCMV6) control (EV), overexpression of STARD3 had no effect on efflux of [^3^H]cholesterol, under basal conditions or in the presence of HDL (50 μg/mL) (Fig. 1E); levels of efflux were comparable with wild type cells. Efflux to apoA-I (10 μg/mL) also remained unaffected by STARD3 overexpression, compared with EV control (*data not shown*). Equally, stable knockdown of endogenous StarD3 had no impact on cholesterol efflux under basal conditions, or in the presence of HDL, compared with the negative short hairpin plasmid (Fig. 1E); insulin release also remained unaffected by changes of STARD3/StarD3 expression within a 5-fold range (Fig. 1F).

The present study demonstrates that endosomal cholesterol trafficking protein, StarD3, does not appear to be regulated by increases in total cholesterol content, or by marked alterations in cholesterol flux between differing cellular pools in BRIN-BD11 cells. This was a surprising outcome, as expression of STARD3 was clearly sterol-dependent in human THP-1 macrophages (Borthwick et al., 2009) and repressed by genetic obesity in both male and female *fa/fa* rats (Soffientini et al., 2014). The promoter regions of both rat and human StarD3/STARD3 contain putative binding sites for lipid responsive transcription factors, such as peroxisome proliferator activated receptors, retinoid X receptors and SREBPs (Borthwick et al., 2009), while the marked increases in cholesterol biosynthesis observed in response to cholesterol depletion (Table 1) suggest that SREBP-dependent induction of HMG CoA reductase is operating correctly. Thus, it seems that expression of StarD3 protein seems to be dissociated from the cholesterol homeostatic ‘machinery’ in BRIN-BD11 cells.

Further, the StarD3 ligand, lutein (Li et al., 2011), did not alter cellular levels of StarD3, suggesting no changes in stability/degradation of this protein, but subtle changes in sterol metabolism were observed in cells exposed to this carotenoid. Cholesterol efflux to apoAI and HDL were diminished by treatment with lutein, and insulin release moderated so that a significant stimulation was seen in the presence of HDL. Recombinant StarD3 selectively binds lutein with high affinity (K_d_ = 0.45 μmol/L) and in macular retina localises to the cone inner segments and axons, where it is thought to facilitate the uptake of lutein (Li et al., 2011) Studies linking lutein with cholesterol efflux from cells are lacking, but this molecule is transported in HDL and binds to SR-B1 (Kijlstra et al., 2012), so that the modest reductions in efflux noted here could reflect competition for SR-B1 occupancy. Alternatively, increased cellular cholesterol content, as a result of reduced efflux (Fig 1B) or enhanced uptake of cholesterol via SR-B1, may explain the increased insulin release seen in the presence of both lutein and HDL (Fig 1C).

Finally, genetic manipulation of levels of StarD3 protein within a 5-fold range did not impact on either HDL efflux or insulin release from stably transfected BRIN-BD11 cell lines. The former outcome is consistent with the lack of sterol regulation of StarD3 in these cells, but contrasts markedly with findings in other studies. Overexpression of STARD3 increased ABCA1 protein levels, and cholesterol efflux in human THP-1 macrophages (Borthwick et al., 2010) and increased lipidation of exogenous apoA-I in McRH-7777 hepatoma cells (Soffientini et al., 2014). Indeed, StarD3 can access cholesterol in ‘early’ late endosomes and facilitate recycling of this sterol to the plasma membrane, aid the formation of inter-organelle membrane contact sites between late endosomes and the ER, and deliver cholesterol to mitochondria (van der Kant et al., 2013; Alpy et al., 2013); StarD3 is also thought to be involved in actin-mediated dynamics of late endocytic organelles (Holtta-Vuori et al., 2005). One possible reason for the lack of impact resulting from changes in StarD3 expression in insulinoma cells may be that insulin granules are major sites of intracellular cholesterol accumulation, distinct from non-secretory cell types in which cholesterol concentrates in recycling endosomes and trans-Golgi network. This resonates with reports that insulin release is sensitive to inhibition of cholesterol biosynthesis, but not lipoprotein depletion from media (Rutti et al., 2009; Zuniga-Hertz et al., 2015) and suggests that if cholesterol derived from the endocytic pathway does contribute to that found in insulin secretory granules, then it does so in a StarD3 independent manner, either at an earlier stage than that facilitated by StarD3, or via other cholesterol trafficking proteins. Certainly, genetic deletion of the START domain of StarD3 in mice is not associated with development of diabetes or major changes in lipid metabolism (Kishida et al., 2004).

Overall, while enrichment and depletion of cholesterol moderates insulin release from BRIN-BD11 cells, endosomal cholesterol trafficking protein StarD3 does not appear to be regulated by substantial changes in sterol metabolism in BRIN-BD11 cells. Lutein, a StarD3 ligand, decreases cholesterol efflux to ApoA-I and HDL, but genetic overexpression and knockdown of StarD3 does not alter cholesterol metabolism or insulin release. Thus, while StarD3 is expressed in BRIN-BD11 cells, it is not sterol-regulated, and appears functionally distinct from the rest of the cholesterol homeostasis ‘machinery’ which sustains cholesterol at levels required for effective insulin release

## Footnotes

Abbreviations: ABCA1 ( ATP binding cassette transporter A1); ABCG1 (ATP binding cassette transporter G1); ApoA-I (Apolipoprotein A-I); CLC (Cholesterol-lipid concentrate); Gapdh (Glyceraldehyde-3-phosphate dehydrogenase); GSIS (Glucose-stimulated insulin secretion); HDL (High density lipoprotein); LDL (Low density lipoprotein); LDLR (LDL receptor); MCD (Methyl--cyclodextrin); SR-B1 (Scavenger receptor B1); StAR (Steroidogenic acute regulatory protein); StarD3 (StAR-related lipid transfer domain 3); START (StAR-related lipid transfer protein).

The authors are indebted to the Rosetrees Trust for their funding of this project (Ref. M278), to Glasgow Caledonian University (GCU) for providing a PhD studentship to JBP, and to the excellent technical team at GCU for their support.

Joana Borges Pinto and Annette Graham declare that they have no conflict of interest. A portion of this data was presented as a poster to the European Association for the Study of Diabetes (2014).

The article does not contain any studies with human or animal subjects performed by any of the authors.

## Electronic supplementary material

Below is the link to the electronic supplementary material.

Supplementary material 1 (PDF 176 kb)
