# Supplementary material for: The role of endosomal cholesterol trafficking protein, StAR-related lipid transfer domain 3 (StarD3/MLN64), in BRIN-BD11 insulinoma cells
Source: Protein Cell. 2016 Sep 27;7(11):833–8. doi: 10.1007/s13238-016-0315-0 (PMC5084155; doi:10.1007/s13238-016-0315-0)
Supplement: Supplementary file 1 — Supplementary material 1 (PDF 176 kb) [file 13238_2016_315_MOESM1_ESM.pdf]

Letter to the Editor

Supplemental materials

Materials and Methods

Materials Apolipoprotein A-I was purchased from Athens Research and Technology (Georgia, USA). All other materials were from UK companies, or UK suppliers for the companies indicated. Radiochemicals were purchased from Perkin Elmer, antibodies to StarD3, Gapdh and beta tubulin were purchased from AbCAM; sterile tissue culture plastics were from Greiner and tissue culture reagents from Scientific Laboratory Supplies. Short hairpin (Sh) RNA plasmids (SureSilencing shRNA) were purchased from Qiagen and the STARD3 rodent clone (pCMV) from Origene.com via Cambridge Biosciences. Complete™ protease inhibitor cocktail was purchased from Roche, cholesterol lipid concentrate from InVitrogen and methyl-β-cyclodextrin, chemicals, t.l.c.plates and solvents from Sigma Aldrich; the insulin ELISA kits (EZRMI-13K) were from Millipore.

Cell culture Rodent (*Rattusnorvegicus*) insulin secreting cell line BRIN-BD11 (ECACC:10033003), a kind gift from Dr S Patterson (GCU), was maintained in RPMI1640 medium, which contains 11.1mmol/L glucose, supplemented with foetal bovine serum (FBS; 10%, v/v), L-glutamine (2mmol/L) and penicillin-streptomycin (50U ml^{-1} and 50µg ml^{-1}, respectively), at 37 ℃ in a humidified atmosphere of 95% air and 5% CO_2.Cells were plated at a density of 30,000-40,000 cells cm^{-2} for 24h, except where indicated; transfection with 2µg of empty vector (pCMV), pCMV_STARD3 or shRNA plasmids were achieved using AmaxaNucleofector-II (kit VCA-1003; protocol G-010). Stable populations were selected using G418 (400 µg ml^{-1}).
Cholesterol enrichment of BRIN-BD11 cells was achieved by treatment with cholesterol-lipid concentrate (CLC) at the dilutions indicated for 1h; cholesterol depletion by use of methyl-β-cyclodextrin at the concentrations indicated for 1h; both were followed by a 24h recovery period in serum-free RPMI1640. Lipidation of apoA-I (10µg ml^{-1}; 24h) was assessed in cells labelled with 0.5µCi ml^{-1} [^{3}H]cholesterol, as described (Borthwick *et al.*, 2009; 2010; Soffientini *et al.*, 2014). Lipid synthesis was measured in serum-free RPMI1640 cells containing [1-2 ^{14}C]acetate (0.5 µCi/ml) for 24h (Borthwick *et al.*, 2009; 2010).
Lipid Analyses Cellular lipids were extracted using hexane:isopropanol (3:2, v/v), as detailed (Borthwick *et al.*, 2009; 2010; Soffientini *et al.*, 2014), and extracts dried under N_2 before resuspension in isopropanol containing rat liver lipids, and separation by t.l.c. using petroleum ether: diethyl ether: glacial acetic acid (90:30:1 by vol.) as the mobile phase. Lipids were identified by comparison with authentic standards, and dpm determined by scintillation counting (Hidex 300SL).
Insulin secretion Insulin release was quantified in Krebs buffer (0.126M NaCl, 2.5mmol/L KCl, 25mmol/L $NaHCO_3$, 1.2mmol/L NaH_2PO_4, 1.2mmol/L $MgCl_2$, 2.5mmol/L$CaCl_2$; pH 7.2) containing 5.6mmol/L glucose, using a Rat/Mouse Insulin ELISA. This physiological concentration of glucose was selected to allow detection of changes in insulin release (positive or negative) caused by changes in cholesterol mass or StarD3 expression.
Protein analyses Protein lysates were prepared in RIPA buffer (25mmol/L TrisHCl pH 7.6, 150mmol/L NaCl, 1% (v/v) NP40, 1% (w/v) sodium deoxycholate, 0.1% (w/v) sodium dodecyl sulphate) supplemented with Complete™ protease cocktail, and separated using 10% (w/v) SDS PAGE gels, transferred to nitrocellulose membranes, and probed using rabbit polyclonal antibodies to StarD3 (1:4000),Gapdh (1:1000) or tubulin (1:1000) as previously (Borthwick *et al.*, 2009; 2010; Soffientini *et al.*, 2014), except that fluorescently-labelled secondary antibodies (Licor) were employed and bands quantified using a Licor Odyssey FC and Image Studio software.

Statistical analysis All values indicate mean±SEM, except where indicated; *n* denotes numbers of independent determinations. Significant (p<0.05) differences were determined using Student's t-test, or repeated measures ANOVA and post-tests, as appropriate.
